# High intravascular tissue factor—but not extracellular microvesicles—in septic patients is associated with a high SAPS II score

**DOI:** 10.1186/s40560-016-0160-5

**Published:** 2016-05-23

**Authors:** Carolin Trepesch, Ramona Nitzsche, Aenne Glass, Bernd Kreikemeyer, Jochen K. Schubert, Sonja Oehmcke-Hecht

**Affiliations:** Institute of Medical Microbiology, Virology and Hygiene, Rostock University Medical Center, Schillingallee 70, 18057 Rostock, Germany; Institute for Biostatistics and Informatics in Medicine and Ageing Research, Rostock University Medical Center, Rostock, Germany; Department of Anaesthesia and Intensive Care Medicine, Rostock University Medical Center, Rostock, Germany

**Keywords:** Sepsis, Tissue factor, Phosphatidylserine, Extracellular vesicles

## Abstract

**Background:**

Sepsis is associated with coagulation abnormalities, and a high content of intravascular tissue factor (TF) may contribute to the development of multisystem organ failure. Circulating microvesicles (MVs) are increased during sepsis and characterized by their phosphatidylserine content. It is unclear whether MVs—as a part of the host response to the infection—are beneficial or rather contribute to systemic complications in sepsis. In the present prospective clinical pilot study, we investigated whether plasma TF and MVs are associated with the risk of multiple organ failure and mortality.

**Methods:**

Thirty patients diagnosed with sepsis, severe sepsis, or septic shock were enrolled and classified as 19 survivors and 11 non-survivors. Blood samples were collected on the day of admission and then daily for up to 2 weeks. MVs and TF were quantified in plasma by ELISA.

**Results:**

Non-survivors had significantly higher TF concentrations on day 3 compared to survivors. Logistic regression analysis revealed that patients with high amounts of TF had significantly increased risk for severity of disease, according to high Simplified Acute Physiology Score II (SAPS II) scores (odds ratio 18.7). In contrast, a higher content of phosphatidylserine-rich MVs were apparently associated with a lower risk for mortality and multiple organ failure, although this was only a trend and the odds ratios were not significant.

**Conclusions:**

This study showed that a high amount of TF in septic patients is significantly associated with increased risk for disease severity, according to a high SAPS II score. Quantification of total MVs in plasma, independent from their cell origin, might be indicative for the outcome of patients in sepsis.

## Background

Sepsis is a serious medical condition caused by an overwhelming immune response to an infection. It is often associated with bacteremia and characterized by systemic signs and symptoms of inflammation [1].

Fifty to 70 % of patients with sepsis develop coagulation abnormalities that range from minor to hyper activation of coagulation. Hyper activation of coagulation frequently causes excess fibrin deposition characterized by simultaneous widespread microvascular thrombosis and profuse bleeding from various sites [2].

The principal initiator of coagulation is tissue factor. Tissue factor (TF) is a transmembrane glycoprotein that triggers blood coagulation via binding factor (F) VIIa and FX with subsequent thrombin generation and fibrin clot propagation. Following vessel injury, TF is exposed to blood and activates the clotting system, which prevents excess blood loss and seals the wound. Under normal conditions, TF is exclusively expressed in vascular and tissue cells that have no direct contact with blood. However, this concept is outdated as evidence supports the presence of intravascular TF, also described as “circulating” or “blood-borne” TF [3]. The cellular source of TF in blood is still a matter of discussion, but it has been concluded that monocytes are the only blood cells that synthesize and express TF [4]. Several groups demonstrated higher levels of intravascular TF in septic patients [5–8].

It is unclear how TF circulates, although it has been described that TF is transported on membrane vesicles. Membrane vesicles are sphere-shaped structures, less than 1 μm of diameter and limited by a lipid bilayer. They can be secreted from all human blood-born cells and are commonly classified according to their formation mechanism and their size. Membrane vesicles with a size between 100 and 1000 nm are called microparticles or microvesicles and shed from the cell plasma membrane, while exosomes are 50–100-nm vesicles that are secreted by cells via exocytosis [9]. The outer surface of membrane vesicles and exosomes is enriched in negatively charged phosphatidylserine (PS), which provides a catalytic surface for many plasma proteins including contact and coagulation factors [10]. Beyond its function in coagulation, PS is also involved in clearance of apoptotic cells and modulation of the immune system [11]. In this paper, the term MVs will be used to designate all types of cell-derived microvesicles that are PS-rich and found in plasma, independent of their size. Depending on cell activation, MVs differ in their composition and function. Elevated levels of MVs have been related to pathological conditions such as bleeding and thrombotic disorders, cardiovascular diseases, cancer, and sepsis [12, 13].

Due to their small size, PS-rich MVs offer an additional phospholipid platform that has approximately 50- to 100-fold more pro-coagulant activity than activated platelets [14]. We recently reported that the number of total PS-rich MVs in plasma was significantly increased in patients suffering from sepsis [15]. Moreover, in another study, we found a significant increase of total MVs in a mouse streptococcal sepsis model, suggesting that their release is an immune response to the infection [16]. As described above, MVs are supposed to be the most important reservoir for intravascular TF; they may transfer and deliver TF to target cells, such as platelets and neutrophils, thereby amplifying and disseminating the pro-coagulant response [10].

These findings raised the question whether MVs—as a part of the host response to the infection—are beneficial or rather contribute to systemic complications in sepsis. In the present prospective clinical pilot study, we investigated the concentration of plasma TF and total MVs in 30 septic patients and evaluated whether both parameters are associated with the risk of multiple organ failure and mortality.

## Methods

### Patients

Thirty patients with sepsis (*n* = 6), severe sepsis (*n* = 4), or septic shock (*n* = 20) were included in this pilot study. Patients were enrolled from the Intensive Care Medicine Unit at University Medical Center of Rostock. The protocol had been approved by our Institutional Ethics committee (A 201151), and informed consent was obtained from the patients or their caring relatives.

All patients were diagnosed according to the guidelines of ACCP/SCCM-Consensus Conference (American College of Chest Physicians/Society of Critical Care Medicine), defined as those patients meeting two of the four systemic inflammatory response syndrome criteria together with an infectious process [17].

The following demographic data were collected at admission in the ICU: age, gender, Simplified Acute Physiology Score II (SAPS II) [10], and Sequential Organ Failure Assessment (SOFA) [12].

### Sampling and methods

Blood samples were collected as patients were diagnosed with sepsis and then daily for up to 7 days, depending on the patient. After 1 week, sampling occurred every second day until day 13. A minimum of two samples and a maximum of 10 samples were derived from a patient. The average study period was 8.6 days. Immediately after blood samples were taken, the blood was centrifuged at 1500×*g*, for 20 min at 21 °C. The resulting platelet-poor plasma obtained was centrifuged again, at 10,000×*g*, for 5 min and 21 °C. The platelet-free plasma obtained after the second centrifugation was stored at −80 °C until analysis.

Total MVs were quantified by a prothrombinase assay that detect PS exposed at the MV surface (ACTICHROME ®MP- Activity ELISA kit, American Diagnostica, USA), according to the instruction of the manufacturer. In this assay, PS-positive MVs bind to Annexin V and expose their PS-rich surface, allowing Factor Xa-Va in the presence of calcium to activate prothrombin into thrombin. The PS concentration is the limiting factor. There is a direct relationship between the PS present on the MVs and the amount of thrombin generation, which is measured via its specific activity on the thrombin substrate. Results were expressed as nanomolar PS by reference to a standard curve established with liposomes of known concentration.

Quantitative tissue factor antigen concentrations in plasma were determined by an enzyme-linked immunoassay (IMUBIND Tissue Factor ELISA kit, American Diagnostica, USA), according to the instruction of the manufacturer. This assay recognizes TF-apo, TF, and TF-VII complexes and is designed such that there is no interference from other coagulation factors or inhibitors of pro-coagulant activity.

Western blot analyses were performed with sheep antibodies against human high molecular kininogen (Affinity Biologicals) and its degradation products as described previously [18].

### Statistics

All data were stored and analyzed using the SPSS statistical package 20.0 (SPSS Inc. Chicago, Illinois, USA). Descriptive statistics were computed for continuous and categorical variables. The computed statistics included mean and standard deviations or median and interquartile range of continuous variables, frequencies, and relative frequencies of categorical factors. Testing for the differences of continuous variables between the study groups was accomplished by the two-sample *t* test for independent samples or the Mann-Whitney *U* test, as appropriate. Test selection was based on evaluating the variables for normal distribution employing the Kolmogorov-Smirnov test.

The logistic regression model was used to assess whether TF or MVs can predict risk of mortality, high SAPS II, and SOFA score. According to their average content of PS and TF, patients were divided into three groups and odds ratios as well as 95 % confidence intervals (95 % CI) were calculated for the outcome of survival, high SAPS II, and SOFA score while one of the groups was used as a reference group. All values resulted from two-sided statistical tests, and *p* ≤ 0.05 was considered to be significant.

### Availability of data and materials

The dataset supporting the conclusions of this article is included within the article. Raw data are available by the corresponding author.

## Results

### Characteristics of patients

The clinical and laboratory data of our patient population are summarized in Table 1. Thirty patients were investigated, 13 (43.3 %) were female and 17 (56.7 %) male. The age ranged between 21 and 84 years; the mean age was 61.03 years. Survivors were younger than non-survivors but not significantly (Table 1). From 30 patients, 19 (63.3 %) survived, whereas 11 (36.7 %) died on ICU. The SAPS II and SOFA scores of non-survivors were significantly higher compared to those of survivors (Table 1). Further on, the activated partial thromboplastin time (aPTT) as a single parameter was significantly prolonged in non-survivors. The aPTT is a marker for the intrinsic coagulation pathway, and the data suggest a consumption of contact factors in non-survivors. However, the prolonged aPTT was not associated with degradation of high molecular weight kininogen in these patients (data not shown).

**Table 1:** Baseline characteristics of patients

	Survivor (*n* = 19)	Non-survivor (*n* = 11)	*p* value
Age (years, mean ± SD)	58.1 ± 16.9	66.1 ± 16.2	0.215
Gender (% of male)	57.9	54.5	–
SAPS II (mean ± SD)	51.65 ± 19.23	73.69 ± 13.85	0.002*
SOFA (mean ± SD)	8.18 ± 3.52	12.29 ± 1.43	<0.001**
PT (s) (median, IQR)	13.96 (13.11–15.13)	14.48 (13.64–1.69)	0.127
aPTT (s) (mean ± SD)	55.18 ± 10.32	70.46 ± 22.92	0.020*
Platelet count (×10) (mean ± SD)	209.3 ± 83.1	148.9 ± 72.9	0.055
White blood cell count (mean ± SD)	14.08 ± 8.61	17.63 ± 9.04	0.293
CRP (mean ± SD)	231.35 ± 99.73	226.79 ± 106.89	0.928
HK (mean ± SD)	0.284 ± 0.032	0.282 ± .021	0.832
Hb (mean ± SD)	5.75 ± 0.65	5.74 ± 0.48	0.952
Procalcitonin (median, IQR)	2.40 (1.36–14.93)	2.73 (1.12–7.99)	0.846
MVs, phosphatidylserine (nM) (mean ± SD)	18.69 ± 6.13	16.63 ± 6.33	0.388
TF (pg/ml) (mean ± SD)	269.75 ± 171.47	325.22 ± 169.82	0.399

Data are expressed as mean ± SD or median and 25 to 75 % interquartile range (IQR) *p < 0.05, **p < 0.001

Other parameters such as platelet count, white blood cell count, and procalcitonin were not significantly different between the two groups (Table 1).

In most of the patients, multiple infectious sources and bacterial isolates were identified (Table 2).

**Table 2:** Sources and microorganism isolates of patients

	Survivors	Non-survivors
Respiratory tract	*Candida albicans 5*	*Candida albicans 2*
	*Candida galbrata 2*	
	*Candida tropicalis 1*	*Candida tropicalis 1*
	*Aspergillus fumagatus 2*	*Strepotoccus anginosus 1*
	*Viridans streptococci 1*	*Staphylococcus aureus 2*
	*Staphylococcus coagulase negative 2*	*Staphylococcus coagulase negative 1*
	*Stenothrophomonas maltophila 1*	*MRSA 1*
		*Enterocabter cloacae 1*
		*Escherichia coli 3*
		*Enterococci 1*
		*Proteus mirabilis 1*
		*Serratia marcescens 1*
Intra-abdominal/pelvis	*Candida albicans 3*	*Candida albicans 3*
	*Candida krusei*	*Candida galbrata 1*
	*Candida tripolis 1*	*Candida tropicalis 1*
	*Staphylococcus capitis 1*	*MRSA 1*
	*Staphylococcus epidermidis 3*	*Staphylococcus epidermidis 2*
	*Staphylococcus warneri 1*	*Staphylococcus haemolyticus 1*
	*Streptococcus anginosus 1*	
	*Enterococci ssp.*	
	*Enterococcus faecalis 4*	*Enterococcus faecalis 2*
	*Enterococcus faecium 4*	*Enterococcus faecium 5*
	*Enterobacter cloacae 1*	
	*Escherichia coli 3*	*Escherichia coli 1*
	*Bacteroides fragilis 3*	
	*Proteus mirabilis 1*	
	*Pseudomonas aeruginosa 1*	*Pseudomonas aeruginosa 1*
	*Anaerob mixed flora 2*	*Anaerob mixed flora 1*
Urinary tract	*Escherichia coli 1*	*Enterococcus faecium 1*
	*Proteus vulgaris 1*	
	*Klebsiella oxytoca 1*	*Klebsiella pneumoniae ssp. 1*
	*Trichosporon asahii 1*	
Heart and vascular system	*Enterococcus faecium 1*	
	*Proprionibacterium acnes 1*	
	*Streptococcus gallolyticus 1*	
	*Staphylococcus epidermidis 1*	
	*Streptococcus alactolyticus 1*	
Blood/catheter	*Proprionibacterium acnes 1*	*Proprionibacterium acnes 1*
	*Staphylococcus epidermidis 1*	*Staphylococcus epidermidis 2*
	*Staphylococcus coagulase negative 2*	*Staphylococcus capitis 1*
		*Staphylococcus hominis 1*
Wound/soft tissue	*Candida albicans 1*	*Escherichia coli 1*
	*Staphylococcus epidermidis 1*	*Staphylococcus aureus 1*
	*Streptococcus pyogenes 1*	

Main sources and organisms detected in septic patients are represented

*MRSA* methicillin-resistant *Staphylococcus aureus*

### Tissue factor

In septic patients, a systemic activation of the extrinsic coagulation pathway by TF may contribute to the development of multisystem organ failure and subsequent mortality [19]. TF circulates in the blood in two different forms: (i) associated with the cellular membrane of white blood cells, platelets, and cell-derived microvesicles and (ii) as a soluble protein lacking the transmembrane domain generated by an alternatively spliced messenger RNA (mRNA) [20]. The ELISA used in the present study detects both forms of TF in plasma [21].

**Fig. 1:**
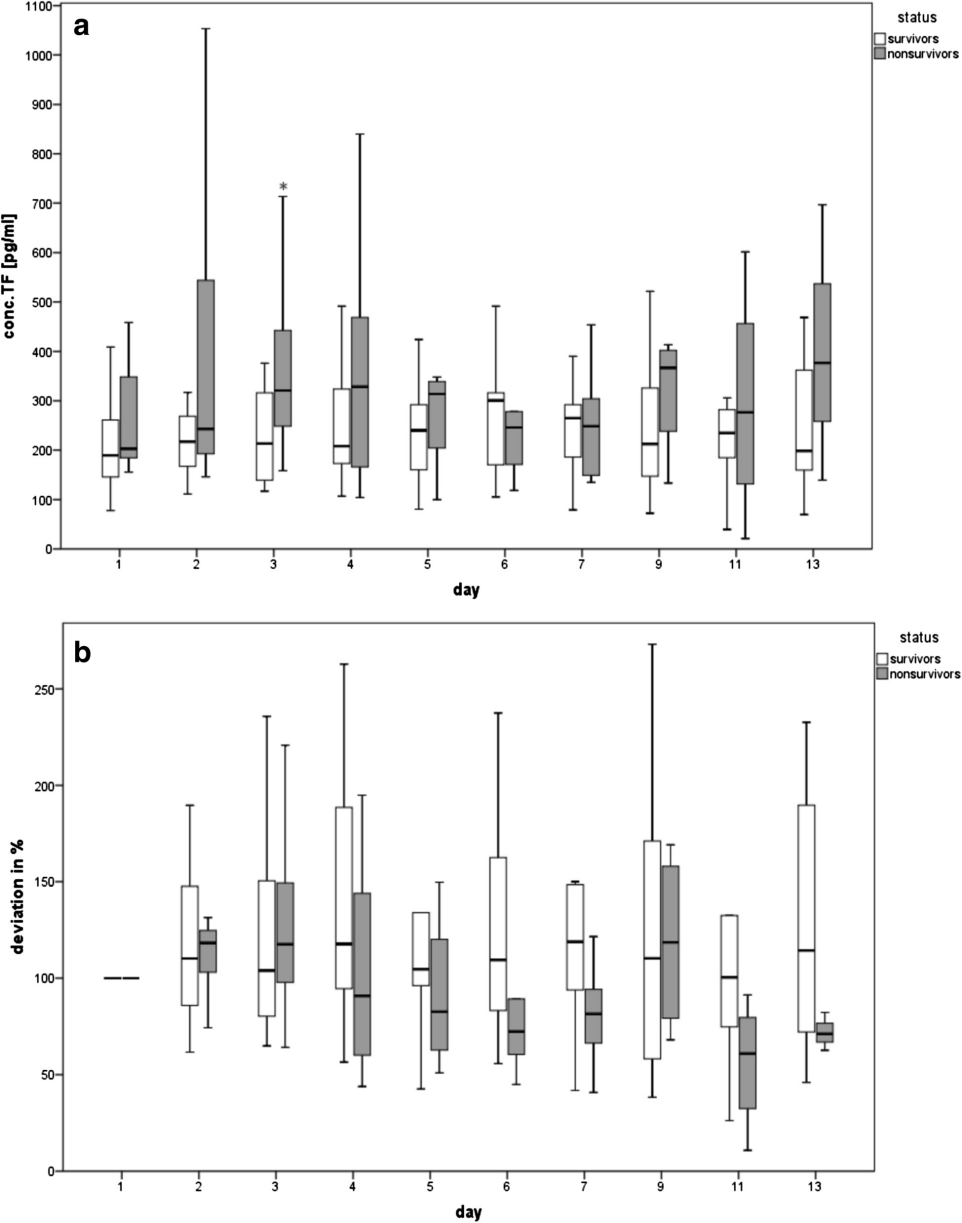
Course of TF antigen in plasma of survivors and non-survivors, depicted as **a** TF concentration (pg/ml) and **b** baseline calculation (%). *p < 0.05 vs. survivors

Figure 1a depicts the TF concentrations of survivors and non-survivors on several days. Hereby, a significant difference between survivors and non-survivors was detected on day 3 (*p* = 0.031), whereby non-survivors had higher concentrations (Fig. 1a). The baseline calculation showed no significant differences (Fig. 1b).

A logistic regression analysis was done. First, the risk of mortality based on TF quantity was calculated, and patients were divided into three groups, (i) patients with high TF concentrations (>300 pg/ml), (ii) patients with middle TF concentrations (220–300 pg/ml), and (iii) patients with low TF concentrations (110–220 pg/ml), who served as the reference group. Patients with middle and high TF concentrations had a 1.5-fold or 2.6-fold higher risk to die, compared to patients with a low TF content (Table 3). Both odds ratios were not significant, but there is a trend towards increased mortality in patients with high TF concentrations.

**Table 3:** TF antigen concentration and the risk of mortality, high SAPS II, or SOFA score calculated by logistic regression analysis

Parameter	Patient group	Odds ratio	95 % CI	Significance
Mortality	TF quantity, middle range	1.52	0.25–9.30	0.65
	220–300 pg/ml			
	TF quantity, high range	2.67	0.39–18.2	0.32
	>300 pg/ml			
SAPS II	TF quantity, middle range	3.20	0.54–18.9	0.20
	220–300 pg/ml			
	TF quantity, high range	18.7	1.56–222.9	0.02*
	>300 pg/ml			
SOFA	TF quantity, middle range	1.46	0.26–8.05	0.67
	220–300 pg/ml			
	TF quantity, high range	5.25	0.70–39.5	0.11
	>300 pg/ml			

*p < 0.05

The patients were divided by their SAPS II score, whereby high SAPS II scores (>60) are accompanied with a higher risk of mortality. The logistic regression analysis was done, and patients with low TF quantity (110–220 pg/ml) were conduced as reference group. Patients with middle TF quantities (220–300 pg/ml) had a 3.2-fold higher risk for a high SAPS II score (Table 3), compared to patients with low TF (110–220 pg/ml). Patients with high amounts of TF (>300 pg/ml) had a statistically significant 18.7-fold increased risk for a SAPS II score above 60 (Table 3), compared to patients with low TF (110–220 pg/ml).

Analogous to the SAPS II score, the patients were divided by their SOFA scores and a value between 11 and 20 was the negative event. The logistic regression analysis showed a 1.46-fold higher risk for patients with middle TF quantity (220–300 pg/ml) for a high SOFA score (Table 3). In patients with high TF (>300 pg/ml), the risk for a SOFA score above 10 (Table 4) was 5.25-fold higher, compared to the reference group (patients with low TF 110–220 pg/ml). Although these odds ratios are not significant, the trend supports the following hypothesis that a high content of intravascular TF seems to be associated with a high risk for mortality.

**Table 4:** MV quantity and the risk of mortality, high SAPS II, or SOFA score calculated by logistic regression analysis

Parameter	Patient group	Odds ratio	95 % CI	Significance
Mortality	MV quantity, low range	3.6	0.62–21.03	0.16
	0–15 nM			
	MV quantity, middle range	1.2	0.15–9.77	0.87
	15–18 nM			
SAPS II	MV quantity, low range	1.7	0.32–8.76	0.54
	0–15 nM			
	MV quantity, middle range	3.5	0.47–25.9	0.22
	15–18 nM			
SOFA	MV quantity, low range	3.5	0.63–19.5	0.153
	0–15 nM			
	MV quantity, middle range	2.7	0.39–18.2	0.32
	15–18 nM			

### PS-positive MVs in plasma

**Fig. 2:**
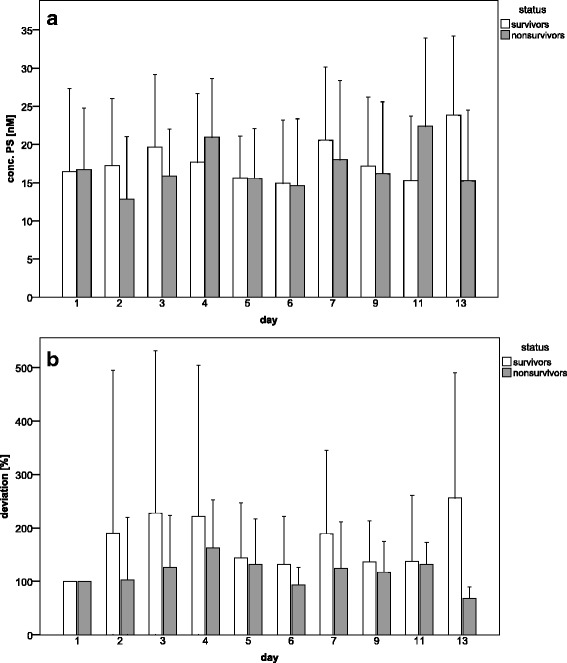
Course of MVs in plasma of survivors and non-survivors, depicted as **a** phosphatidylserine-concentration (nM) and **b** baseline calculation (%)

We measured the concentration of PS-positive MVs in plasma of the septic patients on several days but found no significant differences in MV concentration between survivors and non-survivors (Fig. 2a). The baseline calculation showed no significant differences, although the mean MV quantity in the non-survivor group was lower at all days (Fig. 2b).

To calculate the risk of mortality based on MV quantity, again, a logistic regression analysis was done. Patients were divided into three groups, (i) patients with high MV concentrations (>18 nM) as the reference group, (ii) patients with MV concentrations in the middle range (15–18 nM), and (iii) patients with MV concentrations in the low range (0–15 nM, Table 4). Taken all together, odds ratios implicate that patients with MV concentrations in the middle and low range had a higher risk for mortality or a high SAPS II and SOFA score, when compared to the reference group (Table 4). Although these odds ratios were not significant; the trend supports the hypothesis that a higher content of PS-rich MVs could be associated with a lower risk for mortality.

## Discussion

It is now accepted that TF circulates in the blood: (i) associated with white cells and platelets, (ii) associated with cell-derived MVs, and (iii) as a soluble protein lacking the transmembrane domain generated by an alternatively spliced mRNA [20]. Only the last two forms are detectable in plasma. We found significantly elevated TF concentrations on day 3 in non-survivors of septic patients in comparison with the survivors. This corresponds to the findings in earlier studies comparing survivors with non-survivors. Green et al. found a significant increased TF concentration even on day 3 in children with sepsis [6]. Also Gando et al. showed a significantly higher TF content on several days in non-survivors [21]. Assuming that the SAPS II score reflects severity of sepsis, patients with TF concentrations above 300 pg/ml in our study had a significantly higher risk for a serious course of sepsis. This was supported by a tendency for higher risk of mortality and SOFA score of such patients, though these odds ratios were not significant. Although several studies reported significant higher TF concentrations in plasma of septic patients [5–8], only one study showed a significant correlation between high TF concentrations and increased mortality [6].

An increase in intravascular TF is an indication of tissue factor production from activated monocytes. MVs derived from monocytes express and deliver intravascular TF to sites of pathogen exposure, which initiates immunothrombosis inside a blood vessel [22]. The release of MV-associated TF by monocytes may be therefore regarded as a physiological element of intravascular immunity, to detect and protect against pathogens in the vasculature [23]. However, as we did not found any correlation between TF concentration and total MVs (data not shown), we assume that only a minor part of the circulating MVs contains TF. Another possible reason may be that the ELISA used in the present study detects both MV-associated TF as well as the soluble form of TF.

The pro-coagulant activity of MVs has mainly been attributed to TF but MVs also elicit phospholipid-dependent pro-coagulant activity due to the exposure of PS on their membrane [24]. In an earlier study, we found increased concentrations of PS-rich MVs in patients suffering from sepsis compared to healthy persons [15]. As pro-coagulant MVs seem to play a key role in multi-organ dysfunction and septic shock, it was, therefore, tempting to speculate that a high amount of total PS-rich MVs in septic patients is associated with a higher risk for mortality. Surprisingly, the data of our present study suggest the opposite; a higher content of total MVs was associated with a trend to lower risk for mortality in septic patients, which is in contrast to TF. One could speculate that the MVs are trapped in the organs; therefore, their amount in the circulation is lower. But it should be considered that—behind pathological functions—MVs may be also beneficial at the early stage of sepsis, as they can compensate for some of the host’s systemic reactions [25]. It has been reported that elevated levels of platelet, endothelium-, and leukocyte-derived MVs predict a more favorable outcome in severe sepsis with regard to mortality and organ dysfunction. The authors support the hypothesis that an early increased inflammatory response is associated with improved survival rates [26]. Our recent and actual findings promote this thesis, showing that formation and release of PS-rich MVs are a part of the immune response to bacterial infections [15, 16]. Thus, a high amount of MVs may be a marker of cell activation and inflammation, reflecting a potent inflammatory response, necessary to fight against the infection. A beneficial function for MVs in sepsis was also supported by a study of Mostefai et al., who showed that the total number of circulating and platelet MVs in patients with septic shock was increased. These MVs exert a protective effect against vascular hyporeactivity by maintaining a tonic pressure in septic shock [27]. Also, Guervilly et al. found that high levels of circulating leukocyte MVs are associated with better outcome in acute respiratory distress syndrome [28]. Although the mechanism of protection is not clear, one could hypothesize that the antibacterial effect of MVs, released from human neutrophilic granulocytes, restricts bacterial growth and dissemination during sepsis [29]. In a previous study, we have shown that pro-coagulant MVs can bind to *Streptococcus pyogenes* bacteria and promote clotting, entrapment, and killing of the bacteria in a fibrin network. Thus, an interaction of MVs with bacteria may protect the host [16], which supports the hypothesis above.

A limitation of our pilot study is the relatively small sample size, which reflects typical cases of a Northeast German center for intensive care. This means that the power to detect and label MVs as statistically significant risk factors for mortality was limited. Thus, our data must be validated in an independent larger cohorts of sepsis patients because of the heterogeneity of patients with sepsis and the fact that disease outcome is related to several baseline characteristics [30, 31]. In the future, elucidation of protective mechanisms of MVs is an emerging challenge to design new therapeutic strategies.

## Conclusions

This study showed that high amounts of intravascular TF in septic patients significantly increased the risk of disease severity, according to SAPS II scores above 60. In contrast, a high amount of PS-rich MVs is not associated with disease severity or mortality.

## Abbreviations

MVs, microvesicles; PS, phosphatidylserine; SAPS II, Simplified Acute Physiology Score II; SOFA, Sequential Organ Failure Assessment; TF, tissue factor
